# Free‐Base Octaethylporphyrin on Au(111) as Heterogeneous Organic Molecular Electrocatalyst for Oxygen Reduction Reaction in Acid Media: An Electrochemical Scanning Tunneling Microscopy and Rotating Ring‐Disc Electrode Analyses

**DOI:** 10.1002/smsc.202400294

**Published:** 2024-11-19

**Authors:** Francesco Cazzadori, Alessandro Facchin, Silvio Reginato, Daniel Forrer, Christian Durante

**Affiliations:** ^1^ Department of Chemical Sciences University of Padova via Marzolo 1 35131 Padova Italy; ^2^ Istituto di Chimica della Materia Condensata e di Tecnologie per l'Energia CNR‐ICMATE via Marzolo 1 35131 Padova Italy

**Keywords:** Au(111), electrocatalysis, electrochemical scanning tunneling microscopy, H_2_O_2_, oxygen reduction reaction

## Abstract

The oxygen reduction reaction (ORR) using metal porphyrin catalysts is currently widely explored. Conversely, metal‐free molecular systems are much less investigated, and there is limited information available for molecules such as nonmetalated macrocycles capable of catalyzing the ORR or other small molecules. Herein, the activity and selectivity of a heterogeneous organic molecular electrocatalyst, octaethylporphyrin (H_2_OEP), adsorbed on Au(111) toward ORR in acidic aqueous electrolyte are investigated. Electrochemical scanning tunneling microscopy (EC‐STM) is employed to monitor the molecular layer during the electrochemical process. Additionally, cyclic and linear sweep voltammetries are performed at still and rotating Pt/ring‐H_2_OEP‐functionalized Au(111)/disk electrodes to determine the activity and selectivity of the H_2_OEP monolayer toward ORR on Au(111). Based on EC‐STM and computation analysis, dioxygen electroreduction does not follow an inner‐sphere electron transfer reduction as seen in metal porphyrins, where a preliminary M—O_2_ bond has to form, but it follows an outer‐sphere mechanism involving the precoordination of O_2_ induced by the protonated hydrogen of the macrocycle cavity.

## Graphical abstract

The article clarifies that the adsorption of O_2_ on metal‐free porphyrin is weak and not centered on the molecular ring but on the hydrogen atoms protonating the macrocycle cavity. This weak interaction also accounts for the low but not negligible catalytic activity versus O_2_ reduction, supporting the hypothesis of an outer‐sphere electron transfer mechanism for O_2_ reduction.

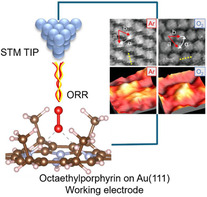

## 1 Introduction

In the context of the growing climate crisis, heterogeneous electrocatalysis of small molecules gives a promising direction toward more efficient electrochemical energy conversion for green energy applications and porphyrin derivatives have proven to excel in this role.^[^ ^1^, ^2^, ^3^ ^]^ Among all the different reactions, the interest in the electrocatalysis of oxygen reduction reaction (ORR) is motivated by its critical importance in the fuel cell and metal–air battery technologies, but also and not less importantly, the oxygen reduction has relevance in biology. The leaking of O_2_^•−^ in biological systems leads to the production of free radicals and cell death and has driven the evolution of superoxide dismutase enzymes.^[^ ^4^ ^]^

ORR occurs at the cathode side of an electrochemical device and possesses a slow kinetic. Platinum‐based materials are known as the best catalysts available at the commercial level.^[^ ^5^, ^6^ ^]^ However, Pt is a critical raw material; therefore, a growing research interest is devoted to metal‐free and platinum group metal‐free catalysts. Among metal‐free electrocatalysts, heteroatom‐doped carbons play a pivotal role. Oxygen is one example of heteroatom that could be easily introduced if not already present in the carbon matrix. Similar to oxygen, nitrogen has a larger electronegativity than carbon, enabling N‐doping to be a method to redistribute the local density of states as well as active sites in the carbon framework. The experimental and theoretical results demonstrated that N atoms facilitated the adsorption of O_2_, thus enhancing the catalytic activity for the ORR. Sulfur is a further common dopant or codopant element for carbon materials, where S facilitates the selectivity versus peroxide production.^[^ ^7^, ^8^, ^9^, ^10^, ^11^, ^12^ ^]^ Organic molecules adsorbed on an electrode support can as well catalyze the O_2_ reduction, mimicking doped carbons. For example, viologens and quinones have good 2e‐ORR activity in alkaline electrolytes, but viologens perform poorly in acidic electrolytes, and quinones do not catalyze 2e‐ORR in acid.^[^ ^13^, ^14^, ^15^ ^]^ Pyrazine derivatives require high overpotential to catalyze 2e‐ORR in acidic environments.^[^ ^16^ ^]^ Recently, 2,2′‐Dipyridylamine was proposed as electrocatalysts for ORR to H_2_O_2_ with an H_2_O_2_ yield of ≈80%, and an onset potential of ≈0.60 V versus reversible hydrogen electrode (RHE) in acidic aqueous electrolyte.^[^ ^17^ ^]^ Besides these few examples, there is a vast organic chemical space still unexplored.

Metal centers coordinated by four nitrogen atoms (MN_4_) pinned on a carbon matrix can be regarded as Pt‐free electrocatalysts and are becoming of particular interest as they showed catalytic activity close to Pt ones.^[^ ^18^, ^19^, ^20^ ^]^ Even if they are regarded as single atom catalyst (SAC), when it comes to identifying the local active site and rationalizing their catalytic activity, there still is a lack of understanding due to the heterogeneity of the catalytic material, which usually consists of a mesoporous carbon matrix composed of a variety of different active sites, from metal and oxide nanoparticles to SACs with different coordination geometries.^[^ ^21^, ^22^ ^]^ For this reason, metal centered macrocycles are studied as a model catalyst allowing to investigate the electrocatalytic properties of the MN_4_ active site by isolating it and precisely tuning the surrounding chemical environment.^[^ ^23^, ^24^ ^]^

**Figure 1:**
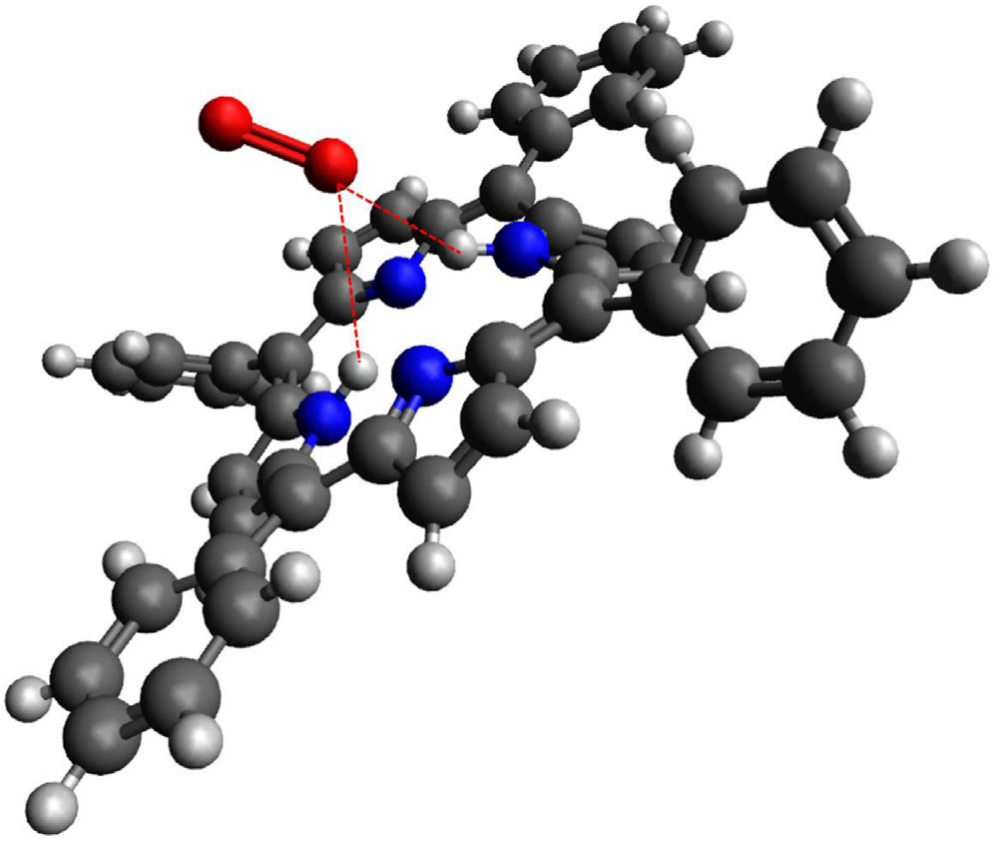
Proposed structure of (H_4_TPP^2+^)‐O_2_ system, ClO_4_^−^ counter anion was omitted for clarity; the averaged O−H distances were calculated to be 2.338 Å, suggesting an interaction with O_2_.^[^ ^26^ ^]^

Metal‐centered macrocycles are commonly employed to investigate both inner‐sphere and outer‐sphere oxygen reduction reactions. In inner‐sphere electron transfer to O_2_, the oxygen molecule must first bind to the active site, typically a metal center, before the electron transfer process occurs. O_2_ is formally reduced, and M is formally oxidized by one electron (M + O_2_ → M–O_2_). This mechanism was clearly demonstrated using scanning tunneling microscopy (EC‐STM) in an electrochemical environment. O_2_ adsorption on the iron single site was clearly visualized before the reduction process. Subsequently, at more reductive potentials, O_2_ was reduced, and the free active site was restabilized.^[^ ^25^ ^]^ On the contrary, in outer‐sphere electron transfer, there is no binding between O_2_ and the active site. Indeed, metal‐free porphyrins were studied as outer‐sphere ORR catalysts because the absence of the metal center eliminates the possibility for an inner‐sphere mechanism. For example, 5,10,15,20‐meso‐tetraphenylporphyrin (H_2_TPP) was found to catalyze, in homogeneous condition, the reduction of O_2_ to H_2_O_2_ dichloroethane/water systems.^[^ ^26^ ^]^ The protonated macrocycles, H_3_TPP^+^ or H_4_TPP^2+^, were proposed to reversibly form unusual adducts with O_2_ **Figure** **1**, with an oxygen–hydrogen distance of 2.338 Å. Evidence of such an adduct are completely missing for heterogeneous systems. The authors calculated a DFT stabilization energy for O_2_ on H_4_TPP^2+^ of 0.112 eV, which is much lower than those calculated for the end‐on O_2_ adduct with Co porphyrin (0.28 eV), which follows an inner‐sphere mechanism.

In this article, we characterize the structure and the reactivity of a metal‐free octaethylporphyrin film supported on an Au(111) (H_2_OEP/Au(111)) surface using a combined approach based on EC‐STM and cyclic voltammetry (CV) in 0.1 M HClO_4_ electrolyte. The aim is to understand whether a heterogeneous catalysis occurs and if O_2_ can indeed follow an inner‐sphere electrochemical reduction by adsorbing on an active site exposed by the macrocycle, such as carbon or nitrogen atoms or rather an outer‐sphere mechanism. EC‐STM has proven to be a powerful tool to study metal porphyrin as model catalysts by coupling with conventional electrochemical measurements, due to its single atom resolution and in situ operating conditions.^[^ ^27^ ^]^ Additionally, when the electrolyte was switched from deaerated to oxygen‐saturated conditions, the activity for the oxygen reduction reaction could be evaluated using EC‐STM by observing the metal‐oxygen coordination.^[^ ^28^, ^29^, ^30^, ^31^, ^32^ ^]^ In this case, the H_2_OEP/Au(111) system is much more challenging because it lacks a clear active site, which is typically present in metal porphyrins. Moreover, the self‐assembly of porphyrins on ideal substrates represents the initial essential step in obtaining a model heterogeneous molecular catalyst suitable for subsequent electrocatalytic investigations. The absence of a metal center makes the self‐assembly process much less favorable. Indeed, acquiring more knowledge about the geometry of porphyrin self‐assembly and mapping potential‐induced phase transitions is crucial not only for electrocatalysis but also for applications in nanoscale electronics and sensing.^[^ ^33^, ^34^, ^35^ ^]^

## 2 Experimental Section

### 2.1 Chemicals, Materials, and Procedures

*N*,*N*‐dimethylformamide (HyPerSolv Chromanorm ‐ VWR Chemicals) was used to prepare 10^−4^ M solutions of the free‐base octaethylporphyrin, which was purchased by Sigma–Aldrich and used without any further purification. Perchloric acid (Fluka TraceSELECT Ultra) was diluted with Millipore Milli‐Q water (specific resistance ≥18.2 MΩ cm, TOC ≤ 5 ppb) to reach a concentration of 0.1 M and used as electrolyte for all the electrochemical experiments. An Au(111) hat‐shaped single crystal from MaTeck was used. Au(111) was functionalized by hanging meniscus method, immersing the surface for 1 min in the porphyrin/DMF solution. The crystal was then mounted in the PEEK electrochemical cell of a home‐built EC‐STM,^[^ ^25^ ^]^ and 0.1 M HClO_4_ was added. The electrolytic solutions were always purged with Ar or O_2_ gas in order to evaluate the system response in the two cases.

The single‐crystal surface must be properly treated before functionalization. Occasionally, it underwent an electrochemical polishing based on anodic oxidation: the gold surface was put in contact with 2 M H2SO_4_, together with a Pt wire as counter electrode, and a polarization of 10 V was applied for 30 s. A brownish layer of Au oxide was formed, and it was removed by immersion in 4 M HCl for 4 min. The surface was finally rinsed. After that, flame annealing was performed with a butane flame.

A 0.25 mm straight tungsten wire was subjected to electrochemical etching in 2 M KOH with a square wave AC generator.^[^ ^36^ ^]^ The obtained tips were rinsed with MilliQ water, and after drying in air they were coated with hot‐melt glue to create an insulating layer on the tungsten, with the aim to prevent Faradaic current at the tip electrode. The very top of the tip will not be covered by the hot glue due to its low curvature radius.

### 2.2 Electrochemical Methods

EC‐STM measurements were performed with a home‐built STM unit developed by Wandelt.^[^ ^37^ ^]^ CV was performed either in situ, with EC‐STM setup, and ex situ, with a standard glass cell. In situ configuration allows to electrochemically characterize the sample even during STM image acquisition, while ex situ setup accounts for better thermostating and degassing (or O_2_ saturation) thanks to a larger volume of electrolyte. The in situ electrochemical measurements were acquired by means of a homemade bipotentiostat coupled with the STM system, while an Autolab PGSTAT204 was employed in the ex situ measurements.

Linear sweep voltammetry (LSV) was carried out on a rotating ring‐disc electrode (RRDE, Pine Instrument: *ϕ* = 5.61 mm Au(111) single crystal disk and a Pt ring), in both Ar‐purged and O_2_‐saturated electrolyte using a Autolab PGSTAT100N bipotentiostat. The measurements were done in a three‐electrode cell thermostated at 25 °C. The RRDE Au(111) disc was used as the working electrode, the Pt ring as detection electrode, and a Pt coil as the counter electrode. Both EC‐STM, CV, and LSV at RRDE measurements are referred to RHE which was freshly prepared before each experiment. It consists in a spiral Pt wire settled to the closed end of a capillary glass tube filled with the electrolyte solution in which H_2_ was directly electrogenerated at the Pt wire via chronoamperometric technique until half of the spiral was filled with gas.^[^ ^38^ ^]^ Glassware was thoroughly rinsed with Milli‐Q water and Piranha solution (1 H_2_SO_4_:1 H_2_O_2_) before introducing any other liquid. STM images were analyzed with WSxM.^[^ ^39^ ^]^

### 2.3 Computational Details

Calculations were performed within the plane‐wave pseudopotential approach as implemented in the Quantum‐Espresso package.^[^ ^40^ ^]^ Cutoffs on wavefunctions and electron density were 30 and 300 Ry respectively. The Perdew–Burke–Ernzerhof for solid approximation to the exchange–correlation functional was employed and atomic cores were described using ultrasoft pseudopotential from the GBRV library.^[^ ^41^ ^]^ Hirshfeld charge analysis^[^ ^42^ ^]^ was performed with a homemade code. Charges were computed using the slab, the $H_{4}{\text{OEP}}^{2+}$ and the $O_{2}$ molecules as fragments in place of single atoms. The Au(111) surface was modeled as a slab with five atomic layers. A $\left(\begin{array}{cc} 6 & 0\\ 1 & 6 \end{array}\right)$ surface cell, containing 36 atoms per layer was adopted. Repeated images along the *z* axis were separated by at least 12 Å of vacuum.

The adsorption energy of $H_{4}{\text{OEP}}^{2+}$ was defined as

(1) $$E_{\text{ads}}=E_{H_{4}{\text{OEP}}^{2+}@\text{Au}\left(111\right)}-\left(E_{H_{4}{\text{OEP}}^{2+}}+E_{\text{Au}\left(111\right)}\right)$$

The binding energy of $O_{2}$ was defined as

(2) $$E_{\text{bind}}=E_{O_{2}–H_{4}{\text{OEP}}^{2+}@\text{Au}\left(111\right)}-\left(E_{O_{2}}+E_{H_{4}{\text{OEP}}^{2+}@\text{Au}\left(111\right)}\right)$$

with the porphyrin adsorbed at a bridge site. A similar equation was used for the binding energy of the $H_{2}O$ molecule.

## 3 Results and Discussions

Obtaining successful molecular self‐assembly is not trivial because it depends on the geometry of the single molecule, the type of substrate, the type of electrolyte, the functionalization procedure, and the applied potential.^[^ ^43^ ^]^ Several metal‐octaethylporphyrins were deposited on an Au(111) electrode, forming a well‐ordered monolayer and, in some cases, the self‐assembly geometries have shown to depend on the applied potential. However, to our knowledge, no studies were published on the free‐base octaethylporphyrin deposited on Au(111). The only known example of a free‐base porphyrin, namely, 5,10,15,20‐tetra(4‐pyridyl)‐21H,23H‐porphine, self‐assembled on Au(111) and studied with EC‐STM in argon‐purged electrolyte (0.1 M H_2_SO_4_), originates from the Borguet group.^[^ ^44^, ^45^, ^46^ ^]^ Borguet & Co. discovered order/disorder transitions and multilayer formation that, respectively, depend on the applied potential. In the first case, this variation induces changes in surface mobility, while in the second case, it modifies the oxidation state of TPyP.

**Figure 2:**
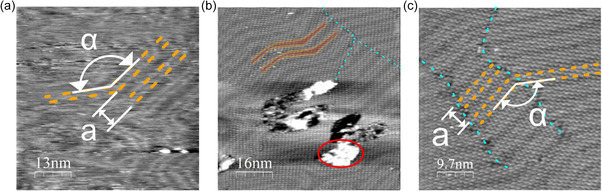
STM images in Ar‐saturated 0.1 M HClO_4_ electrolyte: a) bare Au(111) substrate, where herringbone reconstruction is evidenced by the orange dashed lines: geometric parameters are: *α* = (145 ± 2)°, *a *= (6.3 ± 0.5) nm; b,c) H_2_OEP on Au(111) images at different scales. Domain boundaries are evidenced by azure dashed lines, while herringbone reconstruction is evidenced by the superimposed outline. In (b) a gold island is circled in red while in (c) orange dashed lines follow the herringbone reconstruction with angle *α* = (138 ± 2)° and herringbone periodicity *a* = (6 ± 1) nm.

Before single‐crystal functionalization, STM images of clean Au(111) substrate in 0.1 M HClO_4_ were acquired and are reported in **Figure** **2**a. The clean condition of the system was confirmed by the absence of any additional feature in STM images of the bare gold substrate except of herringbones, terrace steps, and gold islands. The herringbone periodicity was measured by multiple‐profile extrapolation. The obtained value *a* = (6.5 ± 0.5) nm is consistent with the values reported in the literature.^[^ ^47^, ^48^, ^49^, ^50^ ^]^ The angle arising from the change in direction of the soliton is (145 ± 2)° and differs from the expected 120° possibly due to the thermal drift of the piezoelectric scanner.

Images of Au(111) functionalized with H_2_OEP molecules were collected in argon‐purged 0.1 M HClO_4_ and examples of pictures are reported in Figure 2b,c. The functionalized substrate exhibits the usual gold morphology but with periodic features completely covering the gold terraces that can be reconducted to the self‐assembled monolayer of H_2_OEP. Similar patterns were observed for other metal‐centered OEPs on Au(111) in 0.1 M HClO_4_ electrolyte solution.^[^ ^29^, ^51^, ^52^ ^]^ The molecule–substrate interaction accounts for delocalization of π‐electrons in the aromatic ring, which also stabilizes the herringbone reconstruction underneath the molecular layer, even at electrode potential more positive than the corresponding bare surface.^[^ ^53^ ^]^ Moreover, it is interesting to note that the underlying herringbone reconstruction remains visible as a long‐range order pattern. Many studies have reported a modification of the herringbone periodicity after functionalization with molecules, further suggesting that this can be used as a parameter to evaluate the strength of the interaction between self‐assembled organic molecules and the Au(111) substrate. A lifting of the reconstruction indicates a strong interaction, while a weak or middle strength determines a respective perturbation of its periodicity.^[^ ^49^, ^50^, ^54^ ^]^ In the images reported in Figure 2b, herringbone reconstruction periodicity does not change when comparing large‐scale images of the functionalized sample with the bare gold substrate ones (Figure 2a). Bian et al. reported a variation in herringbone periodicity, of ≈1 nm in the module, due to the self‐assembly of 2‐(hydroxymethyl) benzimidazole molecules.^[^ ^50^ ^]^ In our case, we cannot determine with such high precision the herringbone periodicity in the images of functionalized gold because its resolution is reduced by the molecular overlayer. This determines an uncertainty of 1 nm in the quantification of the periodicity value estimated by the standard error of the mean of multiple extrapolated profiles.

The molecular monolayer shows domain boundaries (azure dashed lines in Figure 2b,c), separating domains with the same symmetry but different orientation. Domain boundaries preferentially appear on the herringbone elbows, as already observed for the self‐assembly of 2‐(hydroxymethyl) benzimidazole on Au(111) and they are probably promoted by the low‐coordinated gold atoms at the elbow sites.^[^ ^50^, ^55^ ^]^

**Figure 3:**
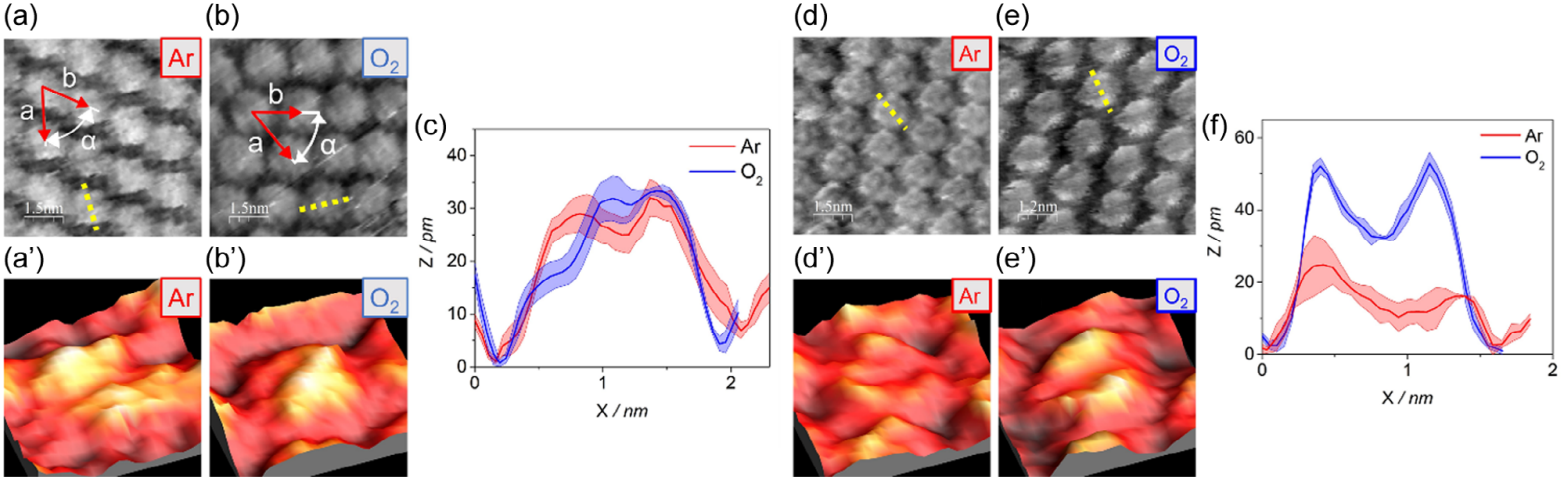
a,b,d,e) High‐resolution EC‐STM images of H_2_OEP on Au(111) in the following conditions: a) Ar purged 0.1 M HClO_4_ electrolyte, *I*_t_ = 1 nA, *U*_b_ = −0.6 V, *E*_app_  = OCP = +0.56 V versus RHE; b) O_2_‐saturated 0.1 M HClO_4_ electrolyte, *I*_t_ = 1 nA, *U*_b_ = −0.6 V, *E*_app_ = +0.56 V versus RHE; d) Ar purged ultrapure water, *I*_t_ = 1 nA, *U*_b_ = −0.6 V, *E*_app_ = OCP = +0.76 V versus RHE; e) O_2_‐saturated ultrapure water, *I*_t_ = 1 nA, *U*_b_ = −0.6 V, *E*_app_ = OCP = +0.7 V versus RHE. a’,b’,d’,e’) 3D view of a region zoomed in from the above image and including only one molecule. c,f) Comparison between the average topographic profile in Ar (red line) and O_2_ (blue line)‐saturated electrolyte extracted from the respective images on the left; more precisely, these plots were obtained by averaging five topographic single‐molecule profiles parallel to the yellow dotted one; the lighter bands result from the standard error of the mean.

High‐resolution images of H_2_OEP on Au(111) in HClO_4_ electrolyte at the OCP are reported in **Figure** **3**. The monolayer is evaluated in terms of lattice parameters and single‐molecule topographic profiles, and these quantities are employed as indicators of the overlayer‐substrate system response to a change of condition, from Ar‐ to O_2_‐saturated electrolyte, and to variations of the applied potential. The H_2_OEP monolayer in deaerated atmosphere is shown in Figure 3a: H_2_OEPs are arranged in a hexagonal symmetry domain with unit base vectors *a* = (1.6 ± 0.1) nm, *b *= (1.7 ± 0.1) nm, and internal angle *α* = (56 ± 5)°. In O_2_‐saturated HClO_4_ electrolyte (Figure 3b), the H_2_OEPs overlayer retains the previously observed hexagonal symmetry with unit base vectors *a* = (1.7 ± 0.1) nm and *b* = (1.8 ± 0.1) nm and internal angle *α* = (57 ± 5)°. To analyze variations in the EC‐STM response at the single‐molecule level when comparing Ar‐ and O_2_‐saturated electrolyte, both the molecular shape and protrusion of molecules were employed. Multiple topographic profiles were extracted and analyzed from different images, keeping the same tunneling conditions and working electrode potential. In the case of HClO_4_ electrolyte (Figure 3a–c,a’,b’), a protrusion of Δ*Z = *30 pm was observed in both atmospheres while a clear variation in the molecular shape arose passing from a more evident central dip in Ar atmosphere to a more plateau‐like shape in O_2_ atmosphere. The same analysis was repeated employing ultrapure water in substitution to HClO_4_ electrolyte. In this case, a clear increment in the molecular protrusion is observed in EC‐STM images taken in O_2_ atmosphere with respect to Ar ones, while the shape undergoes slight variation toward a more pronounced dip (Figure 3d–f,d’,e’). The topographic profiles in Figure 3c,f were calculated by averaging five single‐molecule profiles in the respective top left EC‐STM images; more images were reported in the supporting information to show the negligible variability between different images (Figure S1 and S2, Supporting Information). To further elucidate the effect of the potential over the effect of O_2_ possible adsorption, we have alternatively switched the potential between +0.56 V versus RHE and +0.66 V versus RHE that are the OCP values determined for Ar and O_2_ saturation condition, respectively. As it can be seen from Figure S3 (Supporting Information), a variation from 5 to 10 pm in the molecular height indeed happens when the potential is modified, while the topographic shape is retained.

Metal OEPs are well known to exhibit a notable change in the STM response when the atmosphere is switched from deaerated to O_2_ saturation and, depending on the metal center investigated, it appears as an increment or a decrease of the protrusion in the center of the molecule usually explained with the coordination of molecular oxygen to the metal center.^[^ ^25^, ^29^, ^30^, ^32^, ^56^ ^]^ Therefore, on the base of our findings, EC‐STM analysis suggests that, if any interaction occurs, it is only slight between the molecular oxygen and the free‐base porphyrin molecule adsorbed on the Au(111) substrate. Furthermore, it is worth stressing that no preferential adsorption on peripheral groups or on carbon or nitrogen atoms of the ring was envisaged, which excludes the possibility of these sites to have a clear role in the catalytic reduction of O_2_.

**Figure 4:**
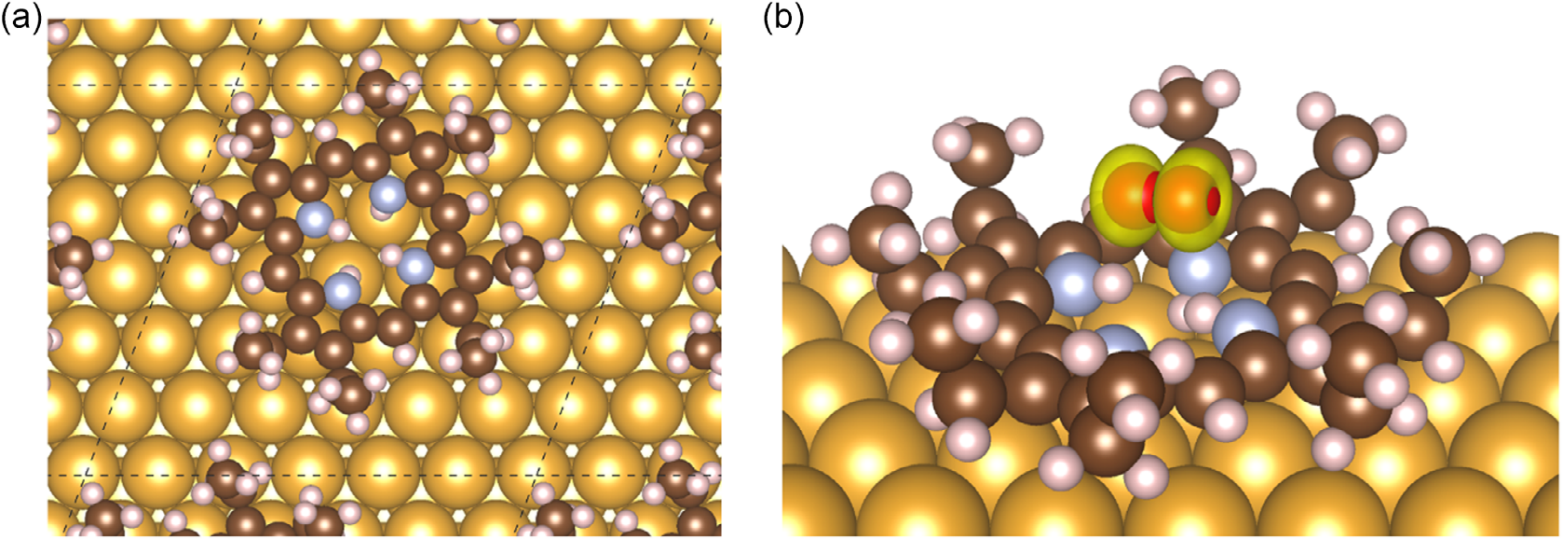
a) Top view of supported $H_{4}{\text{OEP}}^{2+}$, dashed lines represent the surface cell; b) optimized structure of the $O_{2}$ adduct. Color code: C brown, N azure, H white, Au gold. Spin density isosurface is drawn in yellow.

A computational analysis was performed to elucidate the interaction of oxygen with the porphyrin ring adsorbed on an Au(111) surface. The diprotonated $H_{4}{\text{OEP}}^{2+}$ was adsorbed flat on the Au(111) surface with an in‐plane orientation that minimizes intermolecular repulsions **Figure** **4**. The four high‐symmetry adsorption sites, on‐top, face centered cubic, body centered cubic, and bridge, were tested. The system energy was equivalent within 5 × 10^−3^ eV for all sites but the on‐top, whose energy was 0.04 eV higher, shows that there is substantially no preference for a specific adsorption site. In the adsorbed molecule, two opposite N[chemistry single bond solid line]H bonds of $H_{4}{\text{OEP}}^{2+}$ point toward the surface, while the other two point slightly upward. The potential energy surface of the $O_{2}–H_{4}{\text{OEP}}^{2+}$ interaction appears rather flat; binding energies of various configurations are reported in Figure S4 (Supporting Information). O_2_ adsorbs preferentially over the porphyrin core, interacting with the two hydrogen atoms, with binding energy of −0.10 eV and the O−H distance in the range 1.8 ÷ 2.5 Å. For the sake of comparison, equivalent calculations on FeOEP and PtOEP provided an oxygen–metal distance of 1.75 Å and a binding energy of −0.44 and −0.03 eV, respectively.^[^ ^57^ ^]^ The strength of interaction $O_{2}–H_{4}{\text{OEP}}^{2+}$ lies between these two extrema and confirms an interaction with the metal free macrocycle even if proper bond is not formed as in the case of FeOEP. Notwithstanding, the $O_{2}$ bonding energy is fairly weak, especially when compared to the binding energy of the water molecule, which is −0.50 eV. Though there is an attraction between dioxygen and $H_{4}{\text{OEP}}^{2+}$, it is probably too weak to stably replace a water molecule interacting with the adsorbed $H_{4}{\text{OEP}}^{2+}$. These findings support what already made explicit from EC‐STM analysis.

**Figure 5:**
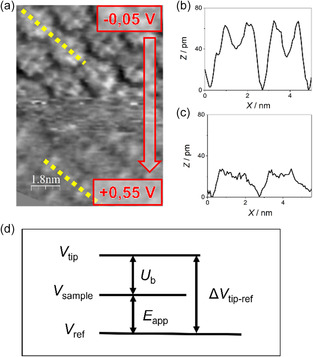
a) High‐resolution image (9 × 12.2) nm in O_2_ sat'd 0.1 M HClO_4_ electrolyte, illustrating the bias effect on molecular STM response. *I*_t_ = 1 nA, *E*_app_ was changed with a 200 mV s^−1^ linear sweep from *E*_app_ = −0.05 V versus RHE in the upper part of the image to *E*_app_ = 0.55 V versus RHE, *U*_b_ changes accordingly from *U*_b_ = 44 mV to *U*_b_ = −544 mV; b,c) topographic profile traced by yellow lines in image (a) in the upper and bottom part, respectively; and d) graphical scheme of different voltages arising in EC‐STM “potentiodynamic” configuration.

The effect of bias voltage is then evaluated by considering the interplay among different voltages arising in the EC‐STM “potentiodynamic” configuration, for which a graphical scheme is shown in **Figure** **5**b. The variation in the applied bias between tip and sample (*U*_b_) should allow the access to different energy states of the probed surface, determining a possible variation in the resolution of the molecular overlayer due to the orbital mediated tunneling effect.^[^ ^58^ ^]^ However, keeping constant *U*_b_ during imaging may not be sufficient to exclude a variation in the local density of states probed while performing “potentiodynamic” measurements, for which a variation in the working electrode potential (*E*_app_) is pursued. In fact, keeping constant *U*_b_, while at the same time sweeping *E*_app_, forces the nominal tip potential to vary with respect to the reference electrode (Δ*V*_tip‐ref_). This could reflect into a variation of the tip work function, implying that a change occurs in the probed energetic states of the sample.^[^ ^59^, ^60^, ^61^ ^]^ Moreover, constantly sweeping Δ*V*_tip‐ref_ (to keep constant *U*_b_) could cause an increment of the Faradaic noise developing on the tip even if its isolation was reasonably good. Therefore, it was rationally decided to keep constant Δ*V*_tip‐ref_ while varying *E*_app_ and, consequently, *U*_b_ changed accordingly with *E*_app_. In Figure 5a, *E*_app_ was swept from −0.05 to 0.55 V with a scan rate of 200 mVs^−1^, while *U*_b_ varied from +0.044 to −0.544 V. As a result, a variation of the molecular resolution occurred, though not impeding the evaluation of the stability of the molecular monolayer.

**Figure 6:**
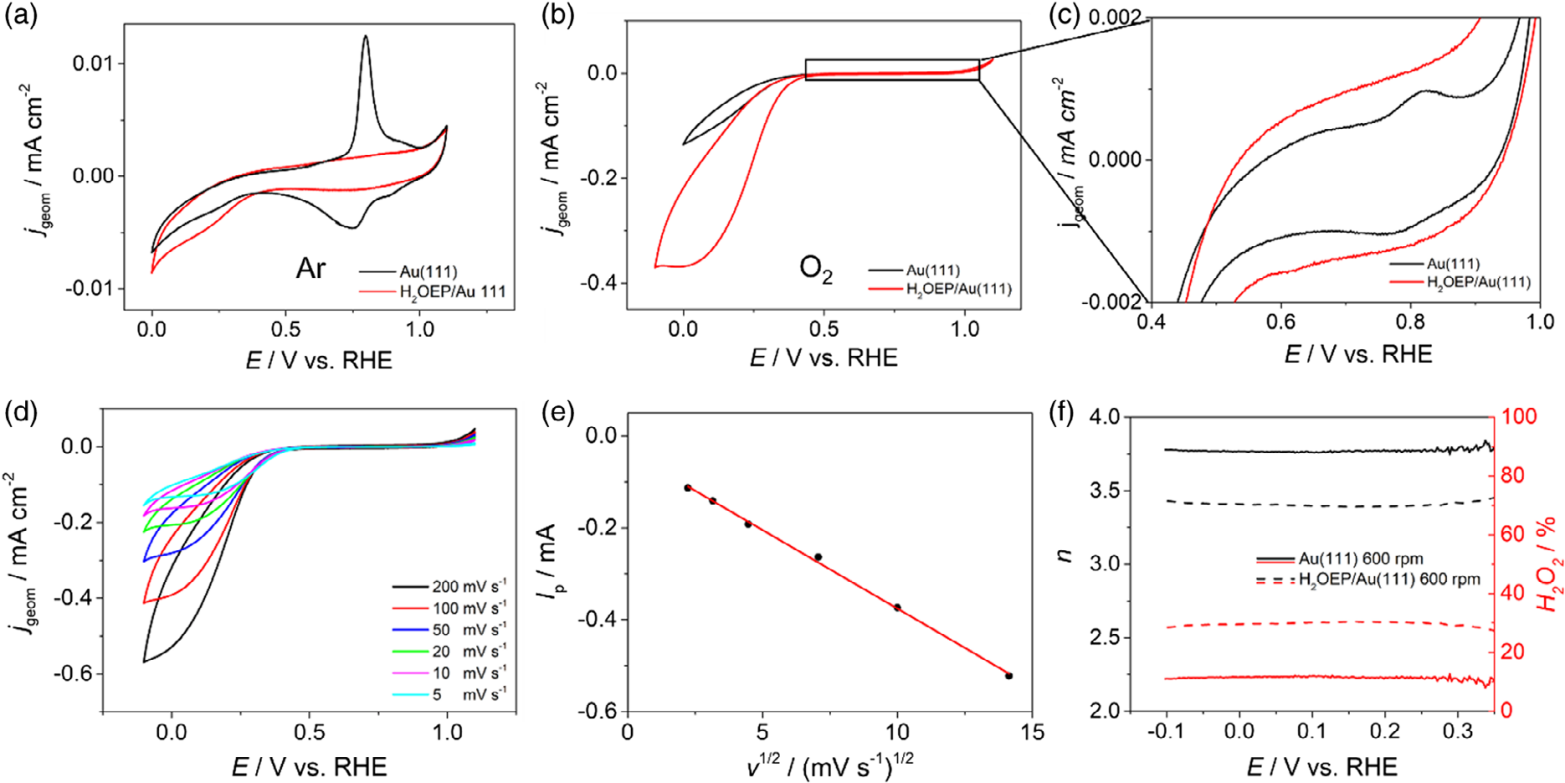
a) CV of bare Au(111) (black line) and H_2_OEP‐functionalized Au(111) (red line) in 0.1 M HClO_4_ aqueous electrolyte saturated with Ar, scan velocity 50 mVs^−1^; b) CV of bare Au(111) (black line) and H_2_OEP‐functionalized Au(111) (red line) in 0.1 M HClO_4_ aqueous electrolyte saturated with O_2_, scan velocity 50 mVs^−1^; c) zoom into the anodic region of CV “(b)”; d) CV of H_2_OEP‐functionalized Au(111) in 0.1 M HClO_4_ aqueous electrolyte saturated with O_2_ at different scan rates; e) linear fit of *I*_peak_[(*v*)^1/2^] data taken from CVs in “(d)”; RRDE results of bare Au(111) (solid lines) and H_2_OEP‐functionalized Au(111) (dotted lines) reporting calculated *n* in black and %H_2_O_2_ in red.

The choice of the working electrode potential window [−0.05; +0.55] V in the “potentiodynamic” experiments was based on the CV response of the H_2_OEP/Au(111) sample. **Figure** **6**a compares CVs in deaerated electrolyte of bare and H_2_OEP‐functionalized Au(111) electrode, respectively. The gold substrate shows a typical reversible peak at positive potentials due to the herringbone reconstruction, which is lifted once the potential is swept toward anodic values and then reconstructed during the cathodic scan. This couple of peaks disappears when the substrate is functionalized with H_2_OEP, suggesting a stabilizing effect exerted by the molecules in favor of the herringbone reconstruction, as already observed in STM images and reported in the literature.^[^ ^53^, ^62^ ^]^ In the cathodic scan, the hydrogen reduction wave sets in at around 0 V for both bare and functionalized substrate, constituting the negative limit of the potential window, while the very small peak anticipating the HER is presumably caused by trace oxygen. Figure 6b,c compares CVs recorded in oxygen‐saturated electrolyte. The observed reduction peak corresponds to ORR, as confirmed by the linear dependence of the peak potential with the square root of scan rate (Figure 6d,e); from the comparison, it is clear that H_2_OEP‐functionalized Au(111) possesses a mild catalytic effect toward ORR, with a positive shift of the onset potential equal to 60 mV with respect to the bare Au(111). The low catalytic activity is in agreement with the low interaction observed by EC‐STM and DFT analysis as well. The selectivity was studied by rotating ring disk LSV (Figure S5, Supporting Information), revealing a threefold increase in %H_2_O_2_ produced by H_2_OEP‐functionalized Au(111) with respect to the bare substrate (Figure 6f). It is important to emphasize, however, that the Faradaic efficiency of the two‐electron process is not 100%. Therefore, the overall process can be considered a mix of both the two‐ and four‐electron processes. Several factors could contribute to this mixed process, such as the quality of the molecular layer, including the possible formation of multilayers or aggregates that can trap H_2_O_2_, leading to its over‐reduction.^[^ ^8^ ^]^ Additionally, the availability of electron density at the Fermi energy of the catalyst, which depends on the arrangement of the molecules, plays a crucial role.^[^ ^63^ ^]^ Finally, the potential presence of secondary active sites capable of rapidly reducing H_2_O_2_ to H_2_O must also be considered.^[^ ^8^ ^]^

This catalytic effect on nitrogen‐doped carbon is commonly justified by the presence of nitrogen atoms, which induces asymmetric charge distribution and so preferential active sites. However, in the present case, it is clear that neither the nitrogen atom nor the adjacent carbon atoms represent a preferential active site; rather, the hydrogen atoms linked to the two nitrogen atoms in the porphyrin ring.^[^ ^64^, ^65^ ^]^

**Figure 7:**
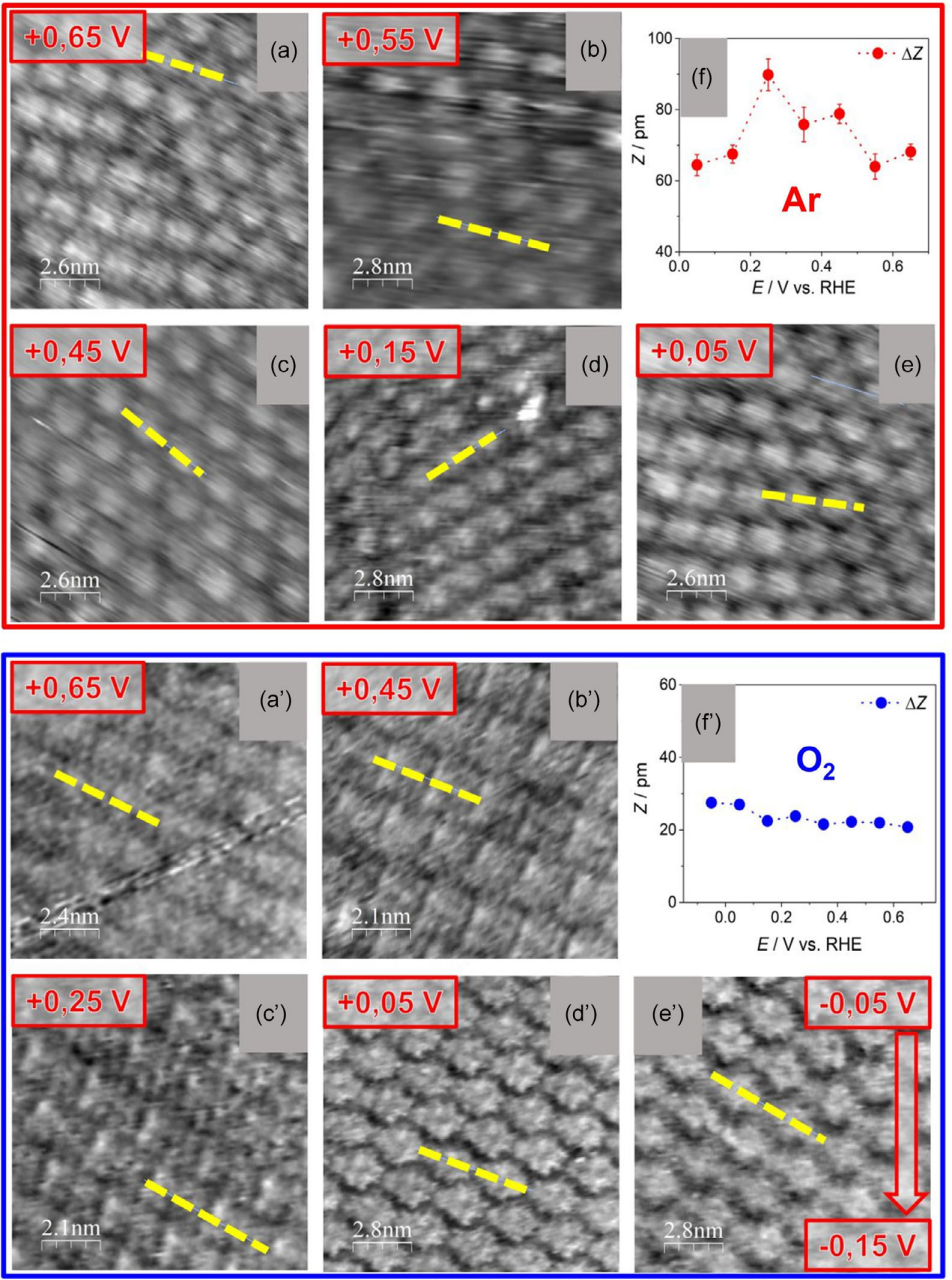
EC‐STM “potentiodynamic” images of H_2_OEP in HClO_4_ 0.1 M electrolyte: in images a–e) the electrolyte is saturated with Ar gas while in images a’–e’) it is saturated with O_2_ gas, *E*_app_ versus RHE are displayed in the red box on top of every image; a) *I*_t_ = 1 nA, *U*_b_ = −590 mV, *E*_app_ = 0.65 V versus RHE; b) *I*_t_ = 1 nA, *U*_b_ = −490 mV, *E*_app_ = 0.55 V versus RHE; c) *I*_t_ = 1 nA, *U*_b_ = −390 mV, *E*_app_ = 0.45 V versus RHE; d) *I*_t_ = 1 nA, *U*_b_ = −90 mV, *E*_app_ = 0.15 V versus RHE; e) *I*_t_ = 1 nA, *U*_b_ = +10 mV, *E*_app_ = 0.05 V versus RHE; a’) *I*_t_ = 1 nA, *U*_b_ = −644 mV, *E*_app_ = 0.65 V versus RHE; b’) *I*_t_ = 1 nA, *U*_b_ = −444 mV, *E*_app_ = 0.45 V versus RHE; c’) *I*_*t*_ = 1 nA, *U*_b_ = −244 mV, *E*_app_ = 0.25 V versus RHE; d’) *I*_t_ = 1 nA, *U*_b_ = −44 mV, *E*_app_ = 0.05 V versus RHE; e’) *I*_t_ = 1 nA, *U*_b_ changed linearly from *U*_b_ + 44 mV in the upper part of the image to *U*_b_ = +144 mV in the lower one, *E*_app_ was swept linearly from *E*_app_ = −0.05 V versus RHE to *E*_app_ = −0.15 V versus RHE; f,f’) are plots of single‐molecule heights (Δ*Z*) versus the applied potential (*E*_app_) in Ar and O_2_‐saturated electrolyte, respectively; Δ*Z* values were obtained averaging a sample of 20 molecules per image extracted from yellow profiles parallel to the one represented in every image.

To evaluate the stability of the molecular monolayer, while catalyzing the ORR, a potential window that starts from the OCP and reaches the ORR peak potential was investigated through “potentiodynamic” measurements **Figure** **7**. The “potentiodynamic” investigation in argon‐ and oxygen‐saturated electrolyte evidenced that the monolayer is stable when the applied potential is varied between +0.65 and +0 V versus RHE. In addition, a statistical analysis of topographic profiles was conducted for every image of the “potentiodynamic” series with the same method employed at the OCP. However, the variations observed of Δ*Z* versus *E*_app_ appear to be not correlated with the ORR process happening at the WE as little to no variation is observed in O_2_ atmosphere, while high oscillations appear in deaerated conditions in a potential range where CV is mostly flat, suggesting that they are mainly caused by the bias potential variation. Therefore, no claims can be made on oxygen adsorption/desorption triggered by the applied potential in the potential window scanned because the Δ*Z* oscillations related to the tip‐sample bias voltage sweep exceed in module the variation of Δ*Z* observed after the exposure to O_2_ gas and associated to porphyrin–O_2_ interaction.

## 4 Conclusions

In this work, the self‐assembly of free‐base octaethylporphyrin on Au(111) substrate in 0.1 M HClO_4_ electrolyte was observed. The molecular overlayer retains the hexagonal geometry of the substrate, stabilizing the herringbone reconstruction. The overlayer unit cell parameters obtained in Ar atmosphere remain unchanged in O_2_ saturation conditions. The single‐molecule topographic profiles taken at the very same applied potential show an almost constant value of heights (Δ*Z*) when the atmosphere is switched from Ar to O_2_, whereas a clear variation in the molecular shape was observed passing from one to the other gas. The same analysis was repeated using ultrapure water, revealing a clear increase in molecular protrusion when EC‐STM images were taken in an O_2_ atmosphere compared to those in an Ar atmosphere, with only slight variations in the molecular shape. However, DFT analysis indicated that even if O_2_ can weakly adsorb over the porphyrin core interacting with the two hydrogen atoms bonded to nitrogen ones, it is highly unlikely for O_2_ to stably displace and replace a water molecule at the center of the porphyrin. Eventually, the molecular monolayers in both atmospheres were monitored while sweeping the applied potential in the region where ORR is expected to occur proving their stability in this potential region because of no adsorption/desorption or phase change phenomena were observed. The molecular height analysis conducted on potentiodynamic images does not allow to claim of any oxygen adsorption/desorption process triggered by the applied potential sweep as the bias voltage‐related oscillations of Δ*Z* exceed in module the variation of Δ*Z* associated to oxygen adsorption. From the electrochemical analysis, the H_2_OEP‐functionalized Au(111) shows a mild catalysis toward ORR and a threefold increment in the percentage of H_2_O_2_ production with respect to the bare Au(111) substrate.

In conclusion, it was elucidated that the adsorption site for O_2_ is weak and not centered on the molecular ring structure, specifically not on the nitrogen atoms or the carbon atoms adjacent to the nitrogen. Instead, the interaction is focused on the hydrogen atom protonating the macrocycle cavity. This weak interaction also accounts for the low catalytic activity, supporting the hypothesis of an outer‐sphere electron transfer mechanism for O_2_ reduction.

## Conflict of Interest

The authors declare no conflict of interest.

## Author Contributions

The manuscript was written through the contributions of all authors. All authors have approved the final version of the manuscript.

## Supporting information

Supplementary Material

## Data Availability

The data that support the findings of this study are available from the corresponding author upon reasonable request.
