# Supplementary material for: Free‐Base Octaethylporphyrin on Au(111) as Heterogeneous Organic Molecular Electrocatalyst for Oxygen Reduction Reaction in Acid Media: An Electrochemical Scanning Tunneling Microscopy and Rotating Ring‐Disc Electrode Analyses
Source: Small Sci. 2024 Nov 19;5(1):2400294. doi: 10.1002/smsc.202400294 (PMC11935223; doi:10.1002/smsc.202400294)
Supplement: Supplementary file 1 — Supplementary Material [file SMSC-5-2400294-s001.pdf]

Supporting Information

Free-base Octaethilporphyrin on Au(111) as Heterogeneous Organic Molecular Electrocatalyst for Oxygen Reduction Reaction in Acid Media: an EC-STM and RRDE analyses.

*Francesco Cazzadori[1], Alessandro Facchin[1], Silvio Reginato[1], Daniel Forrer[1,2] and Christian Durante[1]**

1 Department of Chemical Sciences, University of Padova, via Marzolo 1, Padova 35131, Italy

2 CNR-ICMATE, via Marzolo 1, Padova 35131, Italy

E-mail: christian.durante@unipd.it

S1 Supporting experimental details Pag. 2

S2 Supporting Figures Pag. 3

S3 Supporting Tables Pag. 7

S1 Supporting experimental details

The RRDE analysis was conducted on a Au(111) single crystal substrate in a three electrode cell. The crystal was prepared with flame annealing and functionalized by drop casting of a 0.1 mM H_2OEP solution in N,N-Dimethylformamide (DMF). In Figure S1 linear sweep RRDE voltammetries at 600 rpm rotation rate are reported; the bare Au(111) substrate shows a monotonic increase of ring and disc current, while the H_2OEP functionalized Au(111) reaches a current plateau at negative potentials indicating a diffusive redox process taking place at electrode surface.

The determination of single-molecule protrusion and molecular shape is the result of a statistical analysis of multiple images taken in the same electrolytic interphase, tunneling conditions and working electrode potential.**Error! Reference source not found.** In Figure S1 and S2 are showed the sample of images analyzed for the comparison between Ar and O_2 atmosphere in $HClO_4$ electrolyte and H_2O liquid interphase respectively. An average topographic profile of a single-molecule was calculated for every image with the following method: first, the image was corrected with the WSXM software tools, in particular, a parabolic subtraction for every horizontal line, an equalization to exclude outliers and a gaussian smooth to filter the noise due to the juxtaposition of subsequent scanning lines emerging as horizontal stripes; second, several topographic profiles passing through the center of the molecules are traced along the direction which carries the most amount of information on the overall topographic shape of the molecule, usually they appear with a two lobe simmetry, therefore the direction that cut both the lobe in half is choosen; third, profiles were averaged point by point between the ones extracted in the same image, only the mean profile is reported in the graph with lighter bands representing the standard error of the mean for every averaged point. Figure SFigure S2 **Error! Reference source not found.**

S2 Supporting figures

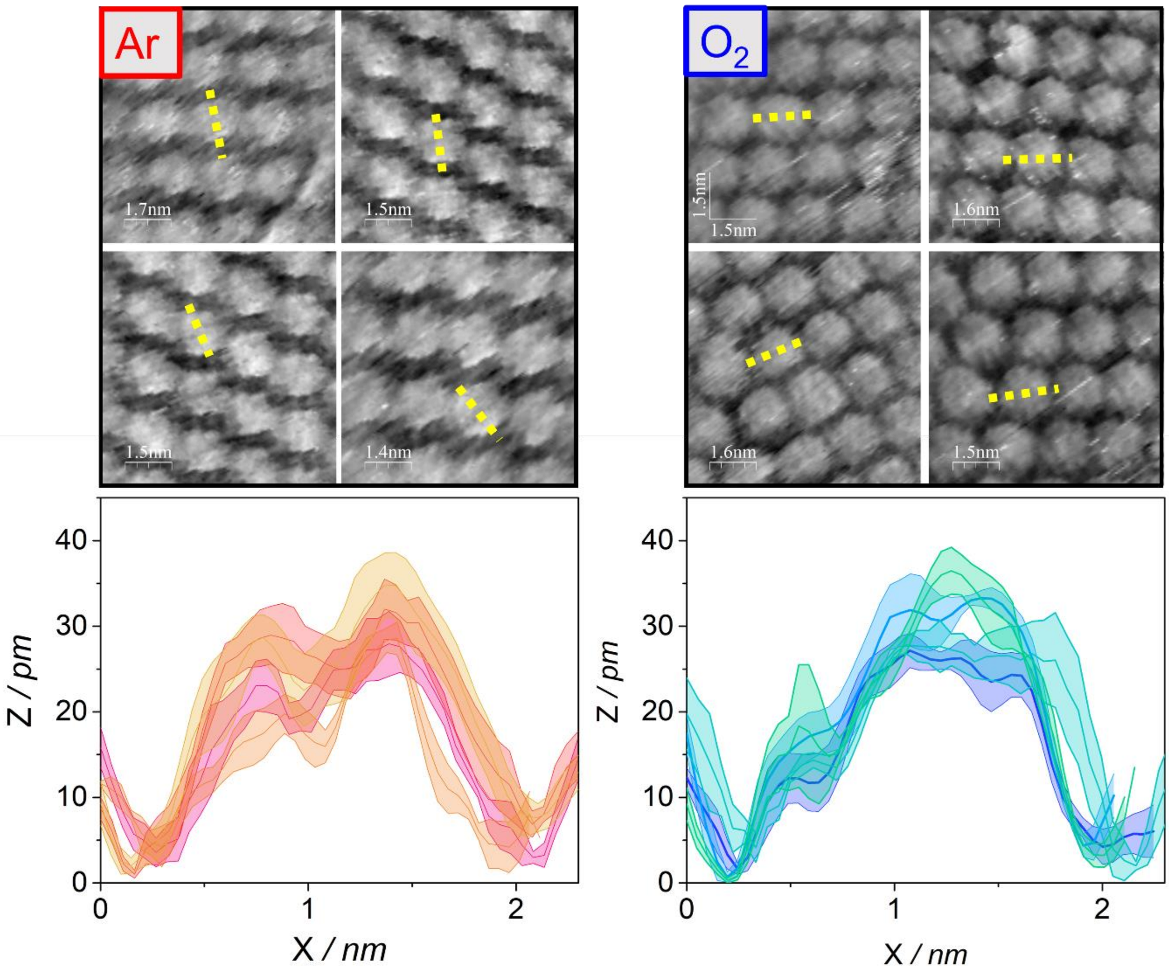

Figure S1. Sample of high-resolution EC-STM image of H_2OEP on Au(111) in 0.1 M $HClO_4$ electrolyte sat'd with Ar (red side) and O_2 (blue side), for every image the tunneling conditions were I_t = 1 nA, U_b = -600 mV and E_{app} = OCP = 0.56 V vs RHE that was monitored to be constant in Ar atmosphere while externally applied in O_2 atmosphere to keep it at the same value. The average topographic profile with standard error of the mean represented as lighter bands is calculated for every image and gathered in the plot below.

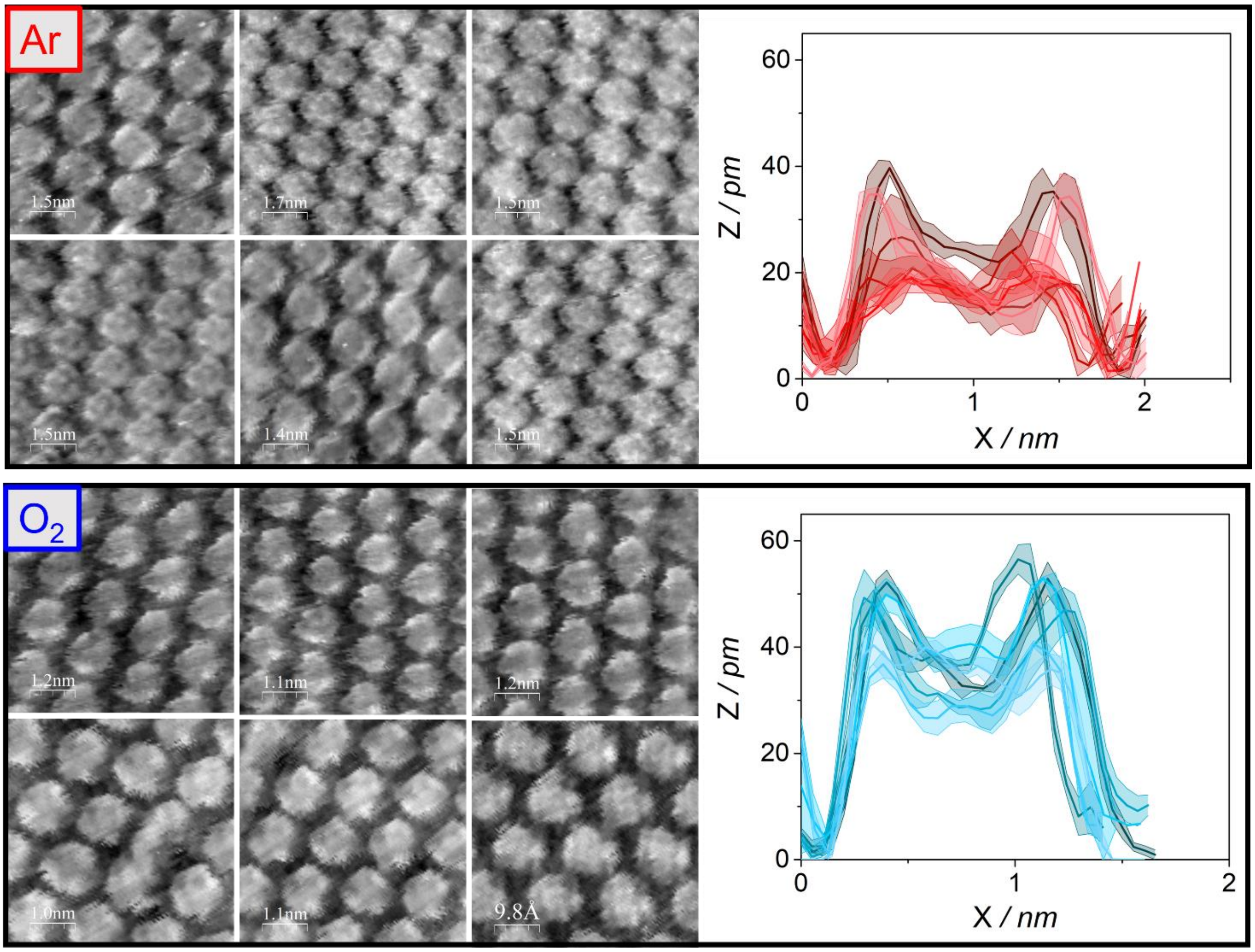

Figure S2. Sample of high-resolution EC-STM image of H_2OEP on Au(111) in H_2O liquid interphase sat'd with Ar (red side) and O_2 (blue side), for every image the tunneling conditions were I_t = 1 nA, U_b = -600 mV, while the OCP was monitored in both atmospheres resulting in a value of E_{app} = OCP = 0.76 V vs RHE in Ar atmosphere and E_{app} = OCP = 0.7 V vs RHE in O_2 atmosphere. The average topographic profile with standard error of the mean represented as lighter bands is calculated for every image and gathered in the plot on the right.

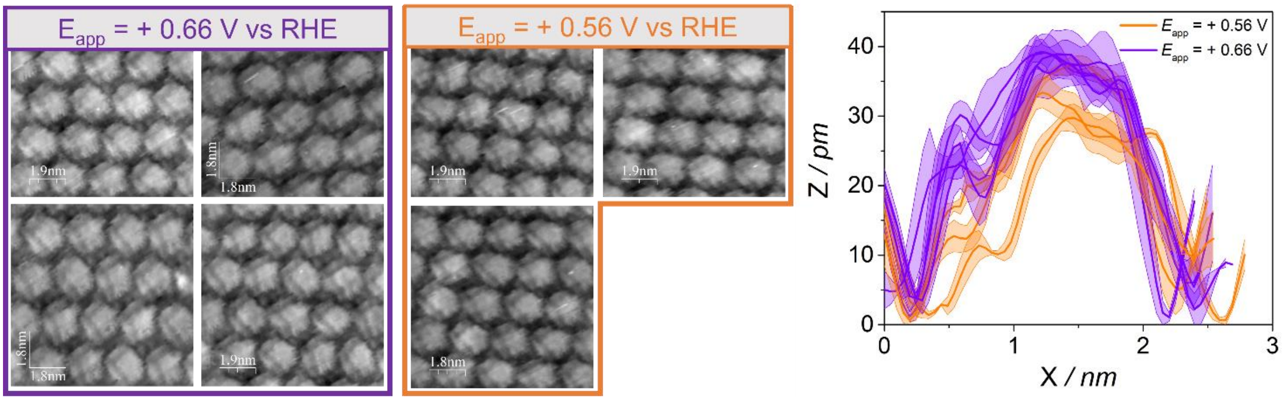

Figure S3 High resolution EC-STM images of H_2OEP on Au(111) in 0.1 M $HClO_4$ electrolyte sat'd with O_2, I_t = 1 nA, U_b = - 600 mV, E_{app} = + 0.56 V vs RHE (orange box and profiles), E_{app}= + 0.66 V vs RHE (violet box and profiles).

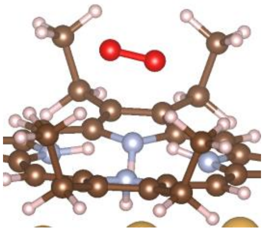	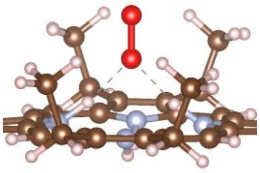	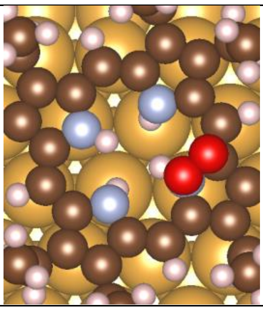
$E_{bind} = -0.10\ eV$<br>$d_{O-H} = 2.48$ Å	$E_{bind} = -0.10\ eV$<br>$d_{O-H} = 1.80$ Å	$E_{bind} = -0.09\ eV$
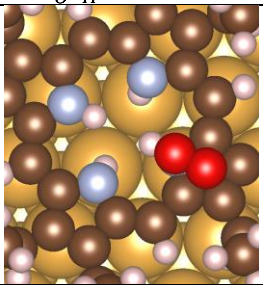	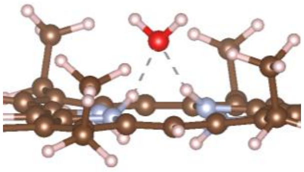	
$E_{bind} = -0.09\ eV$	$E_{bind} = -0.50\ eV$<br>$d_{O-H} = 1.9$ Å	

Figure S4. O_2 and H_2O binding configurations

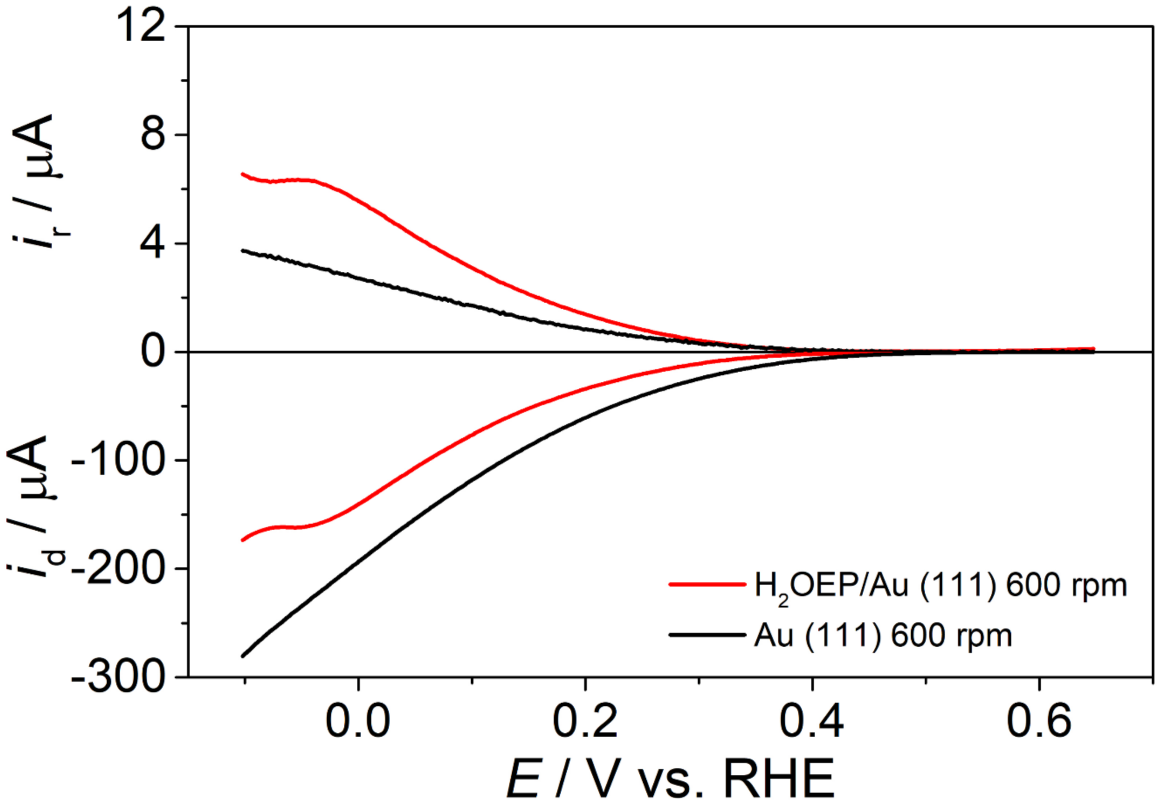

Figure S5. LSV at the RRDE at 600 rpm of Au(111) substrate (black curves) and H_2OEP functionalized Au(111) (red curves). The upper plot shows the ring current while the disk current is reported in the lower one.

S3 Supporting Tables

Table S1. Hirshfeld charges

	$H_4OEP^{2+}@Au(111)$	$O_2 - H_4OEP^{2+}@Au(111)$
O_2		-0.07
H_4OEP^{2+}	1.80	1.84
$Au(111)$	0.20	0.23
